# Long-Term Isoflurane Therapy for Refractory Bronchospasm Associated with Herpes Simplex Pneumonia in a Heart Transplant Patient

**DOI:** 10.1155/2010/746263

**Published:** 2010-12-16

**Authors:** C. Hornuss, M. Firsching, M. Dolch, A. Martignoni, A. Peraud, J. Briegel

**Affiliations:** ^1^Department of Anaesthesiology, Klinikum Grosshadern, Ludwig-Maximilians-University Munich, 81377 Munich, Germany; ^2^Department of Anesthesia and Perioperative Care, UCSF Mount Zion Hospital, University of California San Francisco, 1600 Divisadero Street, C-447, San Francisco, CA 94115, USA; ^3^Department of Neurosurgery, Klinikum Grosshadern, Ludwig-Maximilians-University Munich, 81377 Munich, Germany

## Abstract

A 47-year-old man with a history of heart transplant was admitted after severe traumatic brain injury and seizures. During mechanical ventilation, the patient developed bronchospasm that severely compromised respiratory function that led to cardiac arrest. After resuscitation, application of isoflurane through the Anaesthetic Conserving Device (AnaConDa) in the ICU successfully treated bronchospasm, provided adequate sedation, and enabled appropriate ventilation and diagnostic bronchoscopy. A subsequent bronchoalveolar lavage revealed a high amount of Herpes simplex DNA. Herpes simplex pneumonia was diagnosed and treated with acyclovir. Isoflurane treatment was applied for twelve days total without side effects on renal and cerebral function. The patient recovered quickly after the termination of sedation. At discharge, he was fully awake without focal neurological deficiency and his long-term outcome was excellent. This case demonstrates that isoflurane is a treatment option in life-threatening cases of bronchospasm and a safe option for long-term sedation.

## 1. Introduction

The volatile anesthetic drug isoflurane reduces respiratory resistance and can be applied to treat severe bronchospasm [1, 2]. Though isoflurane is typically administered only for a short period for general anesthesia, it may also be a viable treatment option for severe bronchospasm and for sedation during critical care. However, little experience has been had with long-term treatment of isoflurane (>1 week) or use in patients with traumatic brain injury due to concerns regarding renal toxicity and increased intracranial pressure.

This case report illustrates the successful long-term application of isoflurane in a critically ill patient with several underlying comorbidities.

## 2. Case Report

A fully conscious 47-year-old man was transferred to the hospital after collapse and severe head injury. Twelve years prior to this incident, the patient underwent heart transplantation for treatment of dilatative cardiomyopathy. At admission, computer tomography (CT) revealed traumatic brain injury with an occipital skull fracture, left frontal contusion bleedings, and a traumatic subarchnoid hemorrhage. On the second day of hospitalization, he suddenly suffered from general seizures associated with severe arterial hypotension. After intubation and resuscitation, the patient was treated with anticonvulsants and sedative drugs. A new CT scan excluded any new cerebral injuries or bleedings. 

The patient was then transferred to the ICU. In the presence of fever and clinical and laboratory signs of systemic inflammation, calculated antimicrobial treatment with clindamycin and ciprofloxacin was initiated. Routine clinical, radiological, and microbiological examinations, however, did not reveal any sources of infection. An attempt to reduce sedation to better assess neurological status resulted in severe bronchospasm and impaired lung function as indicated by wheezing and a markedly prolonged expiratory phase. Dynamic hyperinflation occurred and finally led to hypoxia and to cardiac arrest. Cardiopulmonary resuscitation was performed, and lung function was compromised with a paO2/FiO2 ratio of 225 at high airway pressures. Consequently, increasing doses of sedatives became necessary and a combination of midazolam (60 mg · h^−1^), ketamin (200 mg · h^−1^), clondine (120 *μ*g · h^−1^), and sufentanil (75 *μ*g · h^−1^) was needed to protect the patient from any stimuli during positioning and to prevent additional severe events of bronchospasm. We administered the bronchodilators salbutamol (1.5 mg) and ipratropium bromide (500 *μ*g) as aerosole three times per day, magnesium sulfate (20 mmol) intravenously and theophylline (400 mg loading dose over 20 min, then 40 mg · h^−1^) intravenously. Theophylline dose was then adjusted to maintain therapeutic blood levels between 10 and 20 mg · L^−1^ [3]. Both chest X-ray and spiral chest CT scan showed small pleural effusion but again no sign of pneumonia. Repeated microbiological examinations and cultures of tracheal aspirates, urine, or catheter tips did not document any pathogens. A diagnostic bronchoscopy for bronchoalveolar lavage (BAL) was halted due to life-threatening episodes of bronchial obstruction and bronchospasm. 

On day 12 after admission, recurrent episodes of bronchospasm led to an increase of arterial pCO_2_ to 63 mmHg. At this point, still unable to provide adequate sedation, we decided to start inhalative isoflurane therapy with the Anaesthetic Conserving Device Conserving Device (AnaConDa, Sedana Medical, Sundbyberg, Sweden). Inspiratory gas concentration was set to 0.6% which led to Ramsay-score of 5 to 6 points. This allowed appropriate ventilation and adjustment of pCO_2_ to a concentration of 38 mmHg. IV sedation could then be reduced stepwise. Bronchoscopy was repeated successfully and revealed rigid mucus and a highly sensitive bronchial tree that reacted to mild stimuli with bronchoconstruction. Despite this impediment, we performed a bronchoalveolar lavage in the right band, left lower lobes (Segments 7 or 8, resp.). Microbiological cultures of the lavage did not reveal bacterial or fungal growth and polymerase chain reaction (PCR) for cytomegalovirus DNA was also negative. However, PCR for Herpes simplex Typ 1 (HSV-1) virus DNA revealed a high load of 9,300,000 genomic equivalents (ge) mL^−1^. In the throat lavage 19,000,000 ge mL^−1^, HSV-1 was found. 

**Figure 1:**
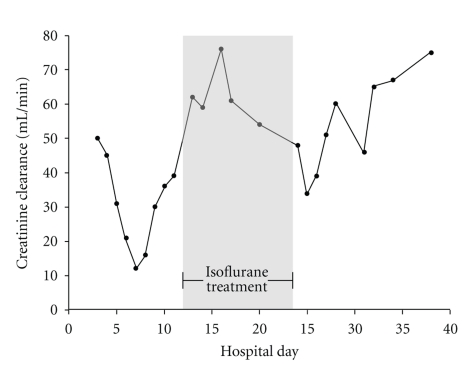
Creatinine clearance of the patient during hospital treatment. Time of isoflurane treatment is indicated by grey background.

Following this discovery, antiviral treatment with acyclovir was initiated. Dosage was adjusted to 2 × 800 mg i.v. according to renal function. Over the next few days, both intensity and frequency of bronchospasm decreased. Six days after the beginning of the antiviral treatment, isoflurane was safely reduced in a stepwise manner and was stopped after a 12-day treatment period. Over the next few weeks, HSV-1 DNA concentrations consistently decreased in throat lavage to 6,200,000 ge mL^−1^ on day 8 and to 250,000 ge mL^−1^ on day 17 of acyclovir treatment. The patient had had a history of impaired renal function and suffered from acute renal failure on day 5 after admission, which made renal replacement therapy necessary from hospital day 5 to 10. However, when isoflurane was initiated, renal function had already recovered (Figure 1), and the patient did not require renal replacement therapy any longer.

Neurologically, the patient recovered quickly after the termination of sedation. At discharge, he was fully awake and had no focal neurological deficiency.

Tracheostomy was performed on hospital day 19 to facilitate weaning from the ventilator. On day 39, the patient was eased off mechanical ventilation. He was transferred to a rehabilitation centre 40 days after hospital admission. At discharge, he was fully awake and had no focal neurological deficiency.

Seven months after the event, the patient was discharged from rehabilitation with no neurological deficiencies. Long-term outcome (as assessed by phone call) was excellent.

## 3. Discussion

In our patient suffering from several morbidities, isoflurane allowed to treat live-threatening bronchospasm and provided long-term sedation. Bronchospasm was most likely caused by HSV-1 pneumonia. While chest X-ray and CT scan did not show alveolar infiltrates typical for bacterial pneumonia, pulmonary function was severely compromised due to airway hypersensitivity. High concentrations of HSV-1 DNA was detected in BAL, but no other sign of bacterial or fungal infection was found after microbiological cultures and DNA analyses. 

Patients treated with immunosuppressive drugs are especially prone to HSV pneumonia and other viral infections. In patients who have HSV pneumonia and are undergoing mechanical ventilation, this is frequently associated with deteriorated lung function and weaning failure, even in the presence of normal X-ray findings [4, 5]. HSV-1 pneumonia is associated with markedly decreased survival during ICU treatment [6–8]. When Linssen et al. examined patients with a positive BAL for HSV-1, they found a considerably increased 14-day mortality rate for patients with an HSV-1 load larger than 10^5^ ge mL^−1^ [7]. In their study, mortality rose from 20% to 41% in patients with a viral load above that cutoff value. In some cases, HSV pneumonia may lead to severe obstructive lung disease. 

Our case demonstrates the successful application of isoflurane in the ICU, for a prolonged period of time, for treatment of refractory bronchospasm. Though our patient had no history of asthma, the underlying HSV-1 pneumonia was associated with severe bronchospasm during mechanical ventilation. Over the course of the disease, intravenous sedative drugs did not achieve adequate sedation level. Especially during positioning and nursing care maneuvers, severe bronchospasm caused life-threatening cardiac depression. The application of bronchodilatory drugs had no beneficial effect.

In patients with traumatic brain injury, control of arterial pCO_2_ levels is of upmost importance and excludes permissive hypercapnia as a treatment option. In this case, the additional application of the volatile anesthetic isoflurane enabled controlled mechanical ventilation and a stable control of pulmonary gas exchange. Finally, isoflurane treatment enabled successful bronchoscopy and BAL which resulted in diagnosis of the underlying infection.

While concerns have been raised about isoflurane possibly causing increased intracranial pressure, it has been shown during neurosurgical procedures that when applied below expired concentrations of 1.2%, isoflurane does not lead to any increase in intracranial pressure [9, 10]. This makes these drugs suitable for sedation in traumatic brain injury. 

Though volatile anesthetics have been shown to be effective in acute asthmatic bronchospasm, data on prolonged application is rare [11]. A common concern against the prolonged use of volatile anesthetics is their potential harm to renal function. During short-term use for surgical procedures, several perioperative studies could not find a deterioration of renal function in patients with pre-existing renal insufficiency after isoflurane anesthesia, but data for long-term application for more than one week is limited [12–15]. Sackey and coworkers compared isoflurane sedation in the ICU to sedation by midazolam in 40 patients for an observation period up to 96 hours [16]. In that study, sufficient sedation using isoflurane was reached without occurrence of hepatic or renal adverse events. In particular, patients receiving immunosuppressive therapy are known to have impaired renal function and decreased creatinine clearance. Our patient with a 12-year history of immunosuppression had experienced his first incident of acute renal failure one year prior to this event during diarrhea, and during our care he had experienced another episode after cardiopulmonary resuscitation which required renal replacement therapy. When isoflurane therapy was started, the patient was already recovering from acute renal failure, and renal replacement therapy had been stopped. Application of isoflurane did not deteriorate renal function and the time course of serum creatinine levels, blood urea, and creatinine clearance during isoflurane therapy can be seen in Figure 1. At discharge, creatinine clearance was in normal range (75 mL/min).

Of note in this case was the use of the AnaConDa system for isoflurane delivery [17]. While most modern ventilators allow the appliance of sophisticated respiratory therapeutic regimes, they usually have no delivery method for volatile anesthetics. The AnaConDa system—which is essentially a modified heat-moisture exchanger—is directly connected to the patient's endotracheal tube and can be combined with any respirator. The device is approved for use with either isoflurane or sevoflurane, which are delivered by a syringe. Sackey and coworkers reported a remarkably lower consumption of isoflurane using the AnaConDa delivery method than from traditional vaporizer techniques and an environmental pollution below the internationally recommended long-term exposure limits which make side effects on ICU personnel unlikely [18].

While isoflurane, in this case, was able to control the patient's pulmonary situation and secure sufficient ventilation, the volatile anesthetic could not be safely withdrawn until the primary source of the bronchospasm was treated. Here, by day 6 of acyclovir antiviral therapy, inspiratory concentrations of isoflurane were able to be reduced stepwise, and isoflurane treatment was able to be stopped one day later. 

To conclude, our case shows the successful long-term application of isoflurane, for a 12-day period, in a patient with severe HSV-1 pneumonia. The application of the volatile anesthetic permitted the therapy of refractory bronchospasm, enabled diagnosis of HSV-1 pneumonia, helped to control pCO_2_ levels, and led to sufficient sedation. The use of isoflurane did not aggravate pre-existing renal insufficiency and was safely performed in a patient with traumatic brain injury.
